# Individuals with knee impairments identify items in need of clarification in the Patient Reported Outcomes Measurement Information System (PROMIS®) pain interference and physical function item banks – a qualitative study

**DOI:** 10.1186/s12955-016-0478-7

**Published:** 2016-05-11

**Authors:** Andrew D. Lynch, Nathan E. Dodds, Lan Yu, Paul A. Pilkonis, James J. Irrgang

**Affiliations:** Department of Physical Therapy, University of Pittsburgh, 229 Bridgeside Point 1, 100 Technology Drive, Pittsburgh, PA 15219 USA; Department of Psychiatry, University of Pittsburgh, Pittsburgh, PA USA; Department of Medicine, University of Pittsburgh, Pittsburgh, PA USA; Department of Psychology, University of Pittsburgh, Pittsburgh, PA USA; Department of Orthopaedic Surgery, University of Pittsburgh, Pittsburgh, PA USA; UPMC Center for Sports Medicine, Pittsburgh, PA USA

**Keywords:** PROMIS, Patient-reported outcomes, Item interpretation, Patient outcome assessment (MeSH), Knee joint (MeSH)

## Abstract

**Background:**

The content and wording of the Patient Reported Outcome Measurement Information System (PROMIS) Physical Function and Pain Interference item banks have not been qualitatively assessed by individuals with knee joint impairments. The purpose of this investigation was to identify items in the PROMIS Physical Function and Pain Interference Item Banks that are irrelevant, unclear, or otherwise difficult to respond to for individuals with impairment of the knee and to suggest modifications based on cognitive interviews.

**Methods:**

Twenty-nine individuals with knee joint impairments qualitatively assessed items in the Pain Interference and Physical Function Item Banks in a mixed-methods cognitive interview. Field notes were analyzed to identify themes and frequency counts were calculated to identify items not relevant to individuals with knee joint impairments.

**Results:**

Issues with clarity were identified in 23 items in the Physical Function Item Bank, resulting in the creation of 43 new or modified items, typically changing words within the item to be clearer. Interpretation issues included whether or not the knee joint played a significant role in overall health and age/gender differences in items. One quarter of the original items (31 of 124) in the Physical Function Item Bank were identified as irrelevant to the knee joint. All 41 items in the Pain Interference Item Bank were identified as clear, although individuals without significant pain substituted other symptoms which interfered with their life.

**Conclusions:**

The Physical Function Item Bank would benefit from additional items that are relevant to individuals with knee joint impairments and, by extension, to other lower extremity impairments. Several issues in clarity were identified that are likely to be present in other patient cohorts as well.

**Electronic supplementary material:**

The online version of this article (doi:10.1186/s12955-016-0478-7) contains supplementary material, which is available to authorized users.

## Background

The Patient-Reported Outcomes Measurement Information System (PROMIS®) is an NIH Roadmap Initiative to improve the efficiency and accuracy of measuring general patient- reported outcomes (PROs) without specific regard to a disease process or body region. The PROMIS initiative created comprehensive item banks to measure the continuum of a single health construct, including physical, mental, and social health. Qualitative and quantitative methods were used to create efficient, precise, valid and responsive short form measures which select items from the larger item banks to tailor the administered PRO to an individual or group [1]. Representative individuals participated in cognitive interviews including qualitative assessment of items in the item banks to identify unclear items and content gaps allowing for item revision to improve understanding [2].

Items can be administered as computerized adaptive tests (CATs), which use fewer items than classical measures and can efficiently and precisely measure health domains [3–5]. Administration with a CAT allows a computer algorithm to select the item that will be most informative, based on the respondent’s status on the domain being measured.

Knee joint impairments caused by acute injuries and degenerative conditions are among the most common musculoskeletal conditions, limiting physical function and causing pain which can interfere with multiple aspects of health [6–10]. It is not clear whether the PROMIS Pain Interference and Physical Function item banks contain items that are clear for individuals with acute and chronic conditions of the knee. The Physical Function item bank is known to include items that are related to the lower extremity, upper extremity, core, and instrumental activities of daily living. It is likely that disease processes and injuries that affect the knee specifically would not impact responses to items that are focused on the upper extremity, however, this has not been explicitly established. The initial quantitative calibration cohort of 21,000 subjects included a general sample, a sample with chronic health conditions, and 900 individuals with OA [11]. Improving items quality by assessing items with the expected end-users is an important aspect of creating a valid item bank, and can result in substantial changes to items [12]. Qualitative interviewing methods have not been implemented in patients with knee joint conditions or other lower extremity impairments [13–16].

Our purpose was to use qualitative methods to identify items in the PROMIS Physical Function and Pain Interference Item Banks that are (1) irrelevant to individuals with knee joint impairments and (2) unclear or otherwise difficult to complete based on item wording [2]. After identifying items that were irrelevant or unclear, our secondary purpose was to suggest revisions to reduce measurement error. Without subjecting the included items to cognitive interviews with patient end-users, the clarity and measurement accuracy of these item banks is unknown. This is part of a larger study intended to provide evidence for interpretation and use of the PROMIS Physical Function and Pain Interference CATs in patients with knee joint impairments.

## Methods

We conducted a Level 2 Conceptual Qualitative Research Study, collecting data from previously identified subgroups of individuals with knee joint impairments (ACL injury, degenerative meniscus tears, and OA) [17]. The parent study (The KneeCAT Study) is an adjunct study to several ongoing, prospective studies of physical function in individuals with knee joint impairments. The design of the parent study prevented our ability to recruit individuals with impairment of the hip or ankle.

**Fig. 1:**
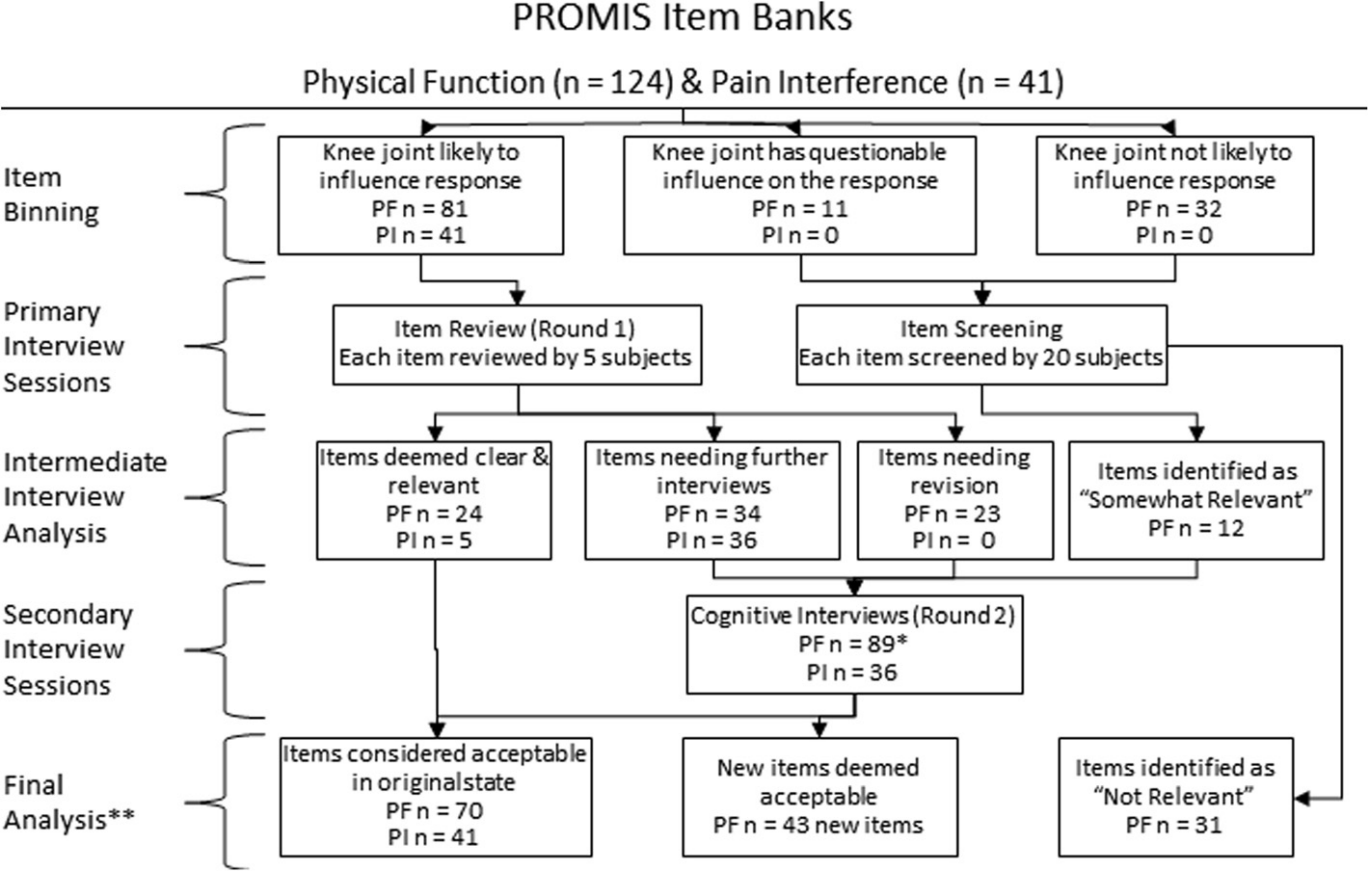
Progression of Study Activities. * Of the 89 Physical Function Items in the Secondary Interview Sessions, 34 were carried over from the Primary Interview Sessions, 12 were introduced from the Item Screening Process, and 43 items were modifications or derivations from existing items. ** Of the 124 items in the Physical Function Item Bank, 70 were considered acceptable, 31 were considered “not relevant”, and 23 were identified as needing revision (see Intermediate Interview Analysis)

Analysis occurred in five phases (Fig. 1). A Binning Process (Phase 1) was completed to sort items by content. Sorted items were used for Primary Interview Sessions and the Item Screening Process (Phase 2). A Preliminary Analysis of interview results was completed (Phase 3) to determine if items needed to be re-written. After the Preliminary Analysis, Secondary Interview Sessions (Phase 4) and a Final Analysis (Phase 5) were completed.

### Item binning

The primary author (ADL) separated the 124 Physical Function and 41 Pain Interference items into bins based on whether the item was likely to be influenced by an impairment of the knee [2]. Categorization was reviewed by the senior author (JJI), and discrepancies were resolved by the study team. All Pain Interference items were placed in bin 1. Items bins included (Additional file 1):

1. Content highly likely to be influenced by knee joint impairment (e.g. climbing stairs).
2. Content somewhat likely to be influenced by knee joint impairment(e.g. being able to reach into a cupboard overhead), and
3. Content not likely to be influenced by knee joint impairment (e.g. buttoning a shirt).

### Participants

Purposive sampling methods (i.e. seeking participants who have specific characteristics) were used to recruit individuals with a knee joint impairment including a broad range of symptom severities and diverse demographics [18]. Individuals with acute and chronic conditions, severe and mild pain, and good and poor function were recruited to participate, with at least two males and females in each category [2]. The study was considered complete when thematic saturation was achieved (i.e. consecutive interviews did not yield novel information that would affect content or interpretation of an item) [19]. Participants provided written informed consent and the study was approved by the Institutional Review Board at the University of Pittsburgh. Three patient-cohorts were recruited: young, athletic individuals with ACL injuries; middle-aged individuals with degenerative meniscus lesions; and older individuals with knee OA.

### Cognitive interview methods

Items from bins 1 and 2 were subjected to mixed-method cognitive interviews following PROMIS Network guidelines [2]. Items were randomly distributed to four blocks that included 30 or 31 items. Each block contained items from both the Physical Function and Pain Interference Item Banks. Items within each block were randomized before each cognitive interview to counterbalance potential effects of fatigue at the end of the interview. Each participant reviewed all items from one block.

During Primary Interview Sessions, participants qualitatively assessed items and response options from a single block. Participants described their interpretation of, opinion of, and response to each item, with prompts from the interviewer as necessary (Table 1). Field notes were recorded to summarize the responses to the interview prompts. Five interviews were completed for each of the four original item blocks, resulting in 20 Primary Interviews. Preliminary Analysis was conducted after the Primary Interview Sessions [20]. Items deemed clear and relevant were removed from further cognitive interviews. Items identified as confusing or unclear in the first 20 interviews were re-written as necessary and included in the Secondary Interview Sessions. Also included in the Secondary Interview Sessions were items in need of further qualitative assessment, newly written items, and items that were identified as potentially relevant in the Screening Process (see below). Items with similar content (e.g. ascending and descending five flights of stairs) were presented in the same block to facilitate direct comparisons. After thematic saturation was achieved, a Final Analysis was conducted.

**Table 1:** Cognitive Interview Prompts and Response Scales

Interview prompts	Content goals
1. Describe the question to me in your own words.	*(Item interpretation, understanding)*
2. What aspects of the item are confusing?	*(Item interpretation, understanding)*
3. What do you think of this item?	*(Item Quality)*
4. How would you go about selecting your answer?	*(Response generation)*
5. What time frame did you base your answer on?	*(Recall time frame)*
6. How would you change this item to improve it?	*(Patient generated improvements)*
7. Is your knee joint relevant to the way you would respond to this item?	*(Relevance of the knee to response)*
Highly Relevant	My knee could affect the way I answer this question.
Somewhat Relevant	My knee could possibly affect the way I answer this question.
Not Relevant	My knee would not affect the way I answer this question.
Is this question important to you?	*(Importance of the item to the individual)*
Highly Important	This question is highly important to understanding how my knee affects my function.
Somewhat Important	This question is somewhat important to understanding how my knee affects my function.
Not Important	This question is not at all important to understanding how my knee affects my function.

During Analysis Sessions after the Primary and Secondary Interview Sessions, field notes were analyzed to determine the general perception of the item and areas of confusion or poor clarity [20]. Participant characteristics were considered during analysis to determine the potential influence of knee impairment (acute or chronic), age, or gender on item response. Items measuring similar content (i.e. walking, household activities) were analyzed together.

### Item screening process

During the Primary Interview Sessions, 20 participants screened the items from bins 2 and 3, and identified whether their knee joint impairment could influence their response to the item. Participants rated the relevance of the item to their knee joint on a three-point scale (Table 1). The Item Screening Process was completed on paper forms and did not include any qualitative assessment or discussion of the items. The Item Screening Process was initially intended to coincide with only the 20 Primary Interviews, however, during the course of the study, we decided to continue administering the Item Screening Forms for the duration of the study through the Secondary Interview Sessions.

During Preliminary Analysis, summary counts for the ratings of item relevance were tallied. It was determined a prior that if 15 of the 20 participants indicated that their knee would not influence their response, the item was deemed to be irrelevant for measuring PRO as it relates to the knee. Items that did not meet the 75 % threshold were considered to be potentially relevant for individuals with a knee impairment and were included in the Secondary Interview Sessions.

## Results

### Participants

Thematic saturation was achieved after 29 interviews, including 20 Primary Interviews and nine Secondary Interviews. Fourteen individuals with acute knee injury (ACL injury, 6 females, ages 15–35) and 15 individuals with chronic conditions (7 meniscus lesions, 4 females; 8 OA, 2 females; ages 42–75) participated.

Ultimately, 70 items in the Physical Function and all 41 items in the Pain Interference Item Bank were considered acceptable without modification. Twenty-three items in the Physical Function Item Bank were considered unacceptable and were re-written into 43 new items, which were considered acceptable by individuals in Secondary Interview Sessions.

### Item screening results

The 20 participants in the Initial Interview Sessions completed the formal Screening Process that was used in the final assessment. Seven of the nine participants in the Secondary Interview Sessions completed the Item Screening Forms. In the transition from the Primary to Secondary Interview Sessions, participants 21 and 22 were inadvertently not administered the screening forms.

Item screening identified 31 items for which the knee was “not relevant” to the item response. These items measured hand and shoulder function, reaching, and basic self-care (Additional file 1). Twelve items dealing with activities in standing were considered to potentially be influenced by an impairment of the knee and were included in the Secondary Interview Sessions. These results were consistent in the seven additional administrations of the Item Screening Form.

### Cognitive interview results

After the Primary Interview Sessions, 24 items from the Physical Function Item Bank and 5 from the Pain Interference Item Bank were determined to be clear, understandable, directly influenced by the knee, and important to individuals with knee impairment (Additional file 2). Thirty-four Physical Function items and 36 Pain Interference items were carried over from the Primary to the Secondary Interviews, respectively. Secondary Interview Sessions included 43 new items. After analysis of all 29 interviews, three aspects of items (themes) were identified which caused confusion. These aspects were not addressed with item revisions.

### Theme 1: “your health”

Twenty-five items in the Physical Function Item Bank begin with the phrase “does your health now limit you in…” to measure multiple aspects of health-related function. Participants indicated the phrase “your health” cued them to think about their whole body and some did not consider their knee joint in such decision-making (Table 2). Respondents suggested that the alternative phrase “your current condition” and that determining which provider was asking for the information (e.g. orthopedic surgeon vs. cardiologist) could be helpful in addressing this issue.

**Table 2:** Interview Comments indicating Variable Interpretations of the Phrase “Your Health”

Item	Item text	Interview comments
PFB54	Does your health now limit you in going OUTSIDE the home, for example to shop or visit a doctor’s office?	*“‘Your health’ makes me think of all kinds of things – blood pressure, dermatological issues, my knee, my Achilles, mental health. The more you know, the harder it is to answer.”*
PFA5	Does your health now limit you in lifting or carrying groceries?	*“My ‘health’ is a full gambit of issues including Crohn’s disease and Parkinson’s.”*
PFB49	Does your health now limit you in going for a short walk (less than 15 min)?	*“The phrase ‘your health’ made me think about total body fitness, but all of my answers were about my knee.”*
PFC35	Does your health now limit you in doing 8 h of physical labor?	*“My knee is a component of my health and is the only big issue right now, but it doesn’t define my health. My health doesn’t limit my ability to do physical labor, but my knee limits what I can do.”*
PFA6	Does your health now limit you in bathing or dressing yourself?	*“‘Your health’ triggered me right to my knee joint. I have a shoulder injury that isn’t bothering me now.”*

### Theme 2: substituting symptom or condition interference for pain interference

Items in the Pain Interference Item Bank begin with “how much did pain interfere with…” followed by a specific situation. Participants without pain often substituted other aspects of their condition that interfered with their life. Substitutions included reduced mobility, having to use crutches, and having to attend rehabilitation or complete exercises (Table 3).

**Table 3:** Interview Comments indicating Variable Interpretations of Pain Interference

Item	Item text	Interview comments
PAININ31	How much did pain interfere with your ability to participate in social activities?	*“The interference is more with my CPM, crutches, and brace than to do with true pain.”*
PAININ53	How often did pain restrict your social life to your home?	*“My lack of mobility is more pertinent when it comes to restricting my social life to home.”*
PAININ56	How irritable did you feel because of pain?	*“In addition to pain, my knees would give out. I would fall and was very irritable and frustrated. I would lash out at simple things due to frustration.”*

### Theme 3: age and gender roles

Participants with chronic impairments commented on the lack of relevance of some items to older individuals, typically dealing with athletic activities (Table 4). Gender roles were identified as influencing item responses. One male participant indicated that he did not do housework. One female indicated that her husband did the lifting of heavy objects.

**Table 4:** Interview Comments Indicating Potential Age Influences on Responses

Item	Item text	Interview comments
PFA1	Does your health now limit you in doing vigorous activities, such as running, lifting heavy objects, participating in strenuous sports?	*“I didn’t even consider the ‘participating in strenuous sports’ part – not in my age bracket.”*
		*“My age and my caution limit me in doing these things.”*
PFA41.a	Are you able to squat like a baseball catcher and get back up?	*“Squatting like a catcher is irrelevant for someone my age.”*
PFC13.a	Are you able to sprint 100 yards?	*“I haven’t sprinted since I was 18. Sprinting is what football players do.”*

### Item revisions based on cognitive interview feedback

Twenty-three items were unclear and in need of revision, resulting in the creation of 43 items. Several areas of poor clarity were identified and addressed to improve item clarity (Table 5). The new items were found to be clear and understandable in Secondary Interview Sessions.

**Table 5:** Examples of Original Items, Interview Comments, and Suggested Revisions

Original item	Interview comments	Suggested revision(s) to item
Does your health now limit you in doing vigorous activities, such as running, lifting heavy objects, participating in strenuous sports?	*“I considered the first two activities (running and lifting heavy objects) more so than strenuous sports.”*	Does your health now limit you in doing vigorous activities including running, lifting heavy objects and participating in strenuous sports?
	*“These activities are not equal.”*	Does your health now limit you in doing vigorous activities?
		Does your health now limit you in participating in sports that you would like to do?
Does your health now limit you in participating in active sports such as swimming, tennis, or basketball?	*“Swimming is different than tennis or basketball. Tennis and basketball involve similar movements, but basketball is more strenuous.”*	Does your health now limit you in activities like swimming, cycling, or golf?
Does your health now limit you in doing strenuous activities such as backpacking, skiing, playing tennis, bicycling or jogging?	*“These activities are not too strenuous. Strenuous activities involve quick movements and long-term running (soccer, basketball). Skiing is the most difficult activity in this item.”*	Does your health now limit you in activities like baseball, softball, or racquet sports?
		Does your health now limit you in playing football, basketball, soccer, or other similar sports?
Does your health now limit you in climbing several flights of stairs?	*“I always have to hold on going down the stairs. If my knees give out, it usually happens going down stairs.”*	Does your health now limit you in going up several flights of stairs?
	*“The pressure associated with climbing stairs made it feel like popping or snapping was imminent.”*	Does your health now limit you in going down several flights of stairs?
Are you able to squat and get up?	*“I don’t do deep knee bends. I thought about sitting in a chair.”*	Are you able to squat like a baseball catcher and get back up?
	*“I do mini-squats in PT, but not full range. This needs to be clearer.”*	Are you able to perform full squats in the gym with resistance?
		Are you able to squat all the way down and get back up?
Are you able to exercise for an hour?	Some subjects did not consider the impact that their injury had on their ability to exercise because they could still exercise with their upper extremity.	Are you able to exercise with your injured body part for an hour?
Does your health now limit you in walking about the house?	Subjects were not clear on the meaning of ‘about the house’ - considered inside and/or outside the home, and may have included stairs	Does your health now limit you in walking on the main floor of your house, not including stairs?

#### Sport and recreation activity categories and descriptions

Physical Function items to measure the ability to complete various sports and daily activities may include multiple specific activities and a general category. Not all of the activities mentioned in the item stem were important to the individual, leading to difficulty in generating a response. Respondents had inconsistent definitions of the modifiers “vigorous” and “strenuous” and work modifiers “heavy” and “moderate”. Replacement items were constructed such that each aspect of the original item was broken out into its own item.

#### Stair negotiation

Most existing stair-related items dealt with stair ascent. Many individuals indicated stair descent was more difficult than ascent, typically due to strength or stability issues. Parallel items were constructed such that each item measuring ascent had a corresponding item measuring descent, with consistent wording (i.e. ‘going up’ and ‘going down’).

#### Exercise

Exercise was identified as important and described as training for fitness or sports, including running, sprinting, cardiovascular training, lifting weights, and performing agility and jumping drills. Participants did not always consider the intensity of the exercise for their lower extremity or substituted the ability to exercise with their upper extremity.

#### Squatting

The original item, “Are you able to squat and get up?” was interpreted inconsistently. Three replacement items were constructed to capture a range of squatting tasks from sports to daily life.

#### Confusing word choices

In the existing items, the term “heavy” is accompanied by the descriptor “10 lb”. Participants indicated a “heavy” item could weigh anywhere from 40 to 200 lb. Three participants identified the ability to carry something heavy as directly relating to quality of life. Replacement items were constructed without a qualifier such that the respondent is able to determine what the term “heavy” means.

Participants identified differences between sprinting, running, and jogging related to the speed and intent of the activity. Jogging was equated to a slow pace, a fast walk, and something people do “just to get out there.” One participant considered the word ‘jog’ to be insulting to her choice of exercise. Sprinting was associated with high-level athletics. Items were constructed to measure each.

Participants identified the word “trousers” as old-fashioned and read the word “stooping” as “stopping”. Participants were inconsistent in interpreting the phrase “about the house” leading to different content influencing responses (e.g. including inside versus outside the house). Replacement items were constructed for clarity and consistency.

### Content categories in the pain interference item bank

One item (PAININ20) asked “how much did pain feel like a burden to you?” confusing participants who associated a ‘burden’ as a physical load to carry. Additionally, phrases such as “take in new information” (PAININ1) and “tasks away from home” (PAININ14) were also considered awkwardly worded by participants.

The Pain Interference Item Bank addresses general areas of physical and social function by using inclusive but indiscriminant terms (e.g. “social activities”, “socializing”, “leisure activities”, “recreational activities”, “simple tasks”, “daily activities”). In defining “social activities”, respondents included activities like dining out and time with family and friends, and excluded participating in sports activities. Some included the amount of walking in social activities, which ranged from no walking to longer walks. Participants included sports when defining “things you usually do for fun” and “leisure activities”, as well as spending time with friends and sedentary activities (going to the movies, watching TV). “Recreational activities” were typically active (sports, walking the dog, playing with kids). One subject indicated “‘recreation’, ‘leisure’, and ‘social’ all blend together, but recreational activities are generally more active.” Overlap was seen between “household chores” and “simple tasks” including housework, yard work, and laundry, with emphasis on cooking, self-care, and basic activities in the “simple tasks” definition. Lastly for “daily” or “usual physical activities”, sports or exercises were included in over half of definitions.

## Discussion

Our purpose was to identify problems with interpretation of items in the PROMIS Physical Function and Pain Interference Item Banks for individuals with knee impairments. Seventy items in the Physical Function and all 41 items in the Pain Interference Item Bank were considered acceptable by participants as originally worded. Two main sources of confusion for participants were identified – generic item wording or item content. Based on the feedback from patient-participants, a number of recommendations were made for the improvement of the PROMIS Physical Function Item Bank. While patients with impairments of other lower extremity structures were not included in this study due to the nature of the funded study design, the results are likely applicable to other regions of the lower extremity with similar impairment (i.e. acute traumatic injury or degenerative conditions). This is especially relevant as many of the issues raised were not specific to the knee joint, but rather to general item clarity or the lower extremity in general. All revisions made to the PROMIS Physical Function Item Bank were general in nature and were not specific to individuals with knee joint impairments.

The vision of the PROMIS Network is to use modern measurement science (e.g., item response theory) to create a state-of-the-art assessment system for self–reported health [21]. The PROMIS measures are written in generic terms and are not intended to be condition- or disease-specific, which can present a challenge for interpretation by patients. The phrase “does your health now limit you in…” may not include the knee joint if the knee is not an important component of a personal definition of health. For items concerning general activity capability (i.e. “Can you exercise for an hour?”), individuals may not consider their injury or condition, but instead think of the accommodations that they make to participate in these activities. Suggested phrases to improve clarity such as “your current condition” instead of “your health” or “with your injured body part” may be useful but raise questions about their consistency with the intent of the PROMIS initiative.

The Pain Interference Item Bank may over-report the influence of pain in individuals limited by condition-specific issues other than pain. Substitution of such an issue is a known bias with item response [2], and individuals post-ACL reconstruction sometimes substituted impairments and surgical restrictions for pain. While these issues interfere with daily life and social participation, they do not do so because of pain. Therefore, it may be prudent to use the PROMIS Pain Intensity scale to identify individuals who have pain before administering the Pain Interference CAT. If Pain Intensity is minimal, the Pain Interference CAT may not be indicated. Incorrectly identifying individuals as having significant pain interference may lead to unnecessary interventions and overestimation of limitations. We are exploring how the Pain Interference CAT performs in individuals who have varying reports of pain intensity and frequency, from minimal to severe. It is also possible that any source of interference may be identified with the Pain Interference Item Bank.

The majority of items were identified as clear. Items measuring basic function (e.g. self-care and activities of daily living) were generally perceived as understandable and relevant to individuals with impairment of the knee. Some items contained confusing words, which is a known issue with legacy items [2]. Items with vague phrases (i.e. “moving about the house”) resulted in variable interpretations and were rewritten. In the Pain Interference Item Bank, terms such as “social activities” and “recreational activities” were not interpreted consistently; however, participants did not identify these as difficult for response. Because the purpose of the item bank is to measure interference and not performance capacity, the variable interpretations of the phrases may not present a significant threat to measurement validity.

Items concerning sport participation were unclear due to multiple content within an item (e.g. activities with varying demands on the knee such as swimming versus cycling). The International Knee Documentation Committee (IKDC) classification is the most commonly used classification related to the knee, grouping activities based on the demand placed on the knee [22]. The most demanding tasks for the lower extremity are sports which require jumping, cutting and pivoting such as football, soccer, and basketball. An attempt was made to reflect the IKDC classification in revised items, which were deemed acceptable. However, the IKDC classification is not pertinent to the upper extremity. Soccer is classified as an IKDC level 1 activity, but would not be identified as demanding for the upper extremity. Overhead sports, such as baseball, softball, or racket sports, place a great demand on the upper extremity, and lesser demands on the lower extremity.

Forty-three items were added to the Physical Function Item Bank to improve clarity or content coverage. The newly created items performed well in cognitive interviews. We are currently administering these items to a sample of individuals with a knee impairment so that they can be calibrated and considered for possible inclusion in the PROMIS Physical Function Item Bank. The PROMIS tools make use of computer adaptive test (CAT) administration of items or short forms of fixed length that can be designed to measure specific ranges of physical function or pain interference. Administration via CAT selects items from the item bank that provide the most information about the individual. CATs have a fixed maximum number of items, thus, adding items to the item bank will not increase the length of the assessment. Rather adding items to the item bank will allow for more precise measurement of a broader range of function with improved ability to discriminate levels of function between individuals.

There is potential for gender and age biases in items as identified by some of the participant comments. Differential item function (DIF) occurs when the probability of an item response is influenced by a characteristic of the individual other than the trait being measured by the item (e.g. age or sex) [23]. In a parallel arm of this study, complete item bank response sets are being collected to assess DIF based on age, sex, and chronicity of condition.

The results of the screening process confirmed the results of Rasch analysis which indicated that items in the Physical Function Item Bank behaved differently for individuals with upper versus lower extremity injuries [24]. The CAT administration method does not currently have any filter in place to select items based on region of impairment, and therefore, an individual with knee OA and an individual with hand OA cannot be distinguished when a CAT version is administered. This was not the intended purpose of the PROMIS Physical Function scale, but this could introduce construct irrelevance if items related to hand and upper extremity function are included in a CAT for individuals with a knee (or hip or ankle) impairment. These items retain importance in measuring physical function from a global perspective. Individuals with knee impairment still require use of their upper extremities, and these items may be appropriate if individuals have concomitant involvement of the upper extremity that also limits physical function. These results indicate a potential need for preliminary items to screen patients and filter items that will introduce unwanted variability to the measurement of physical function if the PROMIS Physical Function Scale is to be used in more specific patient-populations. We plan to further investigate this issue through the assessment of differential item function, and there is a current effort within the PROMIS Network to separate items for measuring upper and lower extremity function with separate banks.

There are some limitations to the results of this study. The participants for this study only represent a subset of individuals with knee joint impairment and are not a representative sample. These participants do not necessarily reflect the views of individuals with hip, ankle, or lower extremity soft tissue impairments. In qualitative research, it is not possible to collect data from a representative sample, and the comments of the participants need to be interpreted with caution. However, as the majority of comments about items were general in nature, it is likely that they apply to other lower extremity impairments. It also is feasible to clarify the instructions for patients to clarify that the PROMIS tools are general in nature.

### Continued research

The revised and added items cannot be included in the item bank as of yet. In a parallel aim of the study, we are collecting responses to the complete item bank and the new and revised items for calibration in a sample of individuals with knee joint impairments. When items are calibrated, they may be included in administrations of computer adaptive tests to ensure that these items contribute to the measurement of physical function. These new items will need to be calibrated for individuals with knee joint impairments to determine if they improve the measurement of physical function. They will also need to be calibrated in a general sample of individuals.

## Conclusion

Caution is needed when implementing the PROMIS Physical Function and Pain Interference tools for individuals with an impairment of the knee. The tools were designed as general measures of physical function and pain interference, and therefore consideration of condition-specific filtering questions may be useful. Generally, the items were well understood by the participants, but some items would benefit from re-wording to improve clarity. The authors have attempted to clarify those items and are pilot testing the new items to calibrate the items within the existing PROMIS Item Banks and to identify DIF.

### Ethics approval and consent to participate

Participants provided written informed consent and the study was approved by the Institutional Review Board at the University of Pittsburgh.

### Availability of data and materials

The dataset supporting the quantitative conclusions of this article is included within the article (and its additional files). The field notes on which the qualitative reports are based will not be made available due to potential issues with breaches of confidentiality.

## Additional file

Additional file 1: Results of Item Screening. (XLSX 16 kb) Additional file 2: Item Results. (DOCX 26 kb)

## Abbreviations

ACL: anterior cruciate ligament
CAT: computer adaptive test
DIF: differential item function
IKDC: international knee documentation committee
OA: osteoarthritis
PAININ: pain interference
PROMIS: Patient Reported Outcome Measurement Information System
PROs: patient- reported outcomes

## References

[1] Hung M, Baumhauer JF, Brodsky JW, Cheng C, Ellis SJ, Franklin JD (2014). Psychometric comparison of the promis physical function cat with the faam and ffi for measuring patient-reported outcomes. Foot Ankle Int.

[2] DeWalt DA, Rothrock N, Yount S, Stone AA, Group PC (2007). Evaluation of item candidates: the promis qualitative item review. Med Care.

[3] Fries JF, Bruce B, Cella D (2005). The promise of promis: using item response theory to improve assessment of patient-reported outcomes. Clin Exp Rheumatol.

[4] Hung M, Baumhauer JF, Latt LD, Saltzman CL, SooHoo NF, Hunt KJ (2013). Validation of promis (r) physical function computerized adaptive tests for orthopaedic foot and ankle outcome research. Clin Orthop Relat Res.

[5] Hung M, Stuart AR, Higgins TF, Saltzman CL, Kubiak EN (2014). Computerized adaptive testing using the promis physical function item bank reduces test burden with less ceiling effects compared to the short musculoskeletal function assessment in orthopaedic trauma patients. J Orthop Trauma.

[6] Cecchi F, Mannoni A, Molino-Lova R, Ceppatelli S, Benvenuti E, Bandinelli S (2008). Epidemiology of hip and knee pain in a community based sample of italian persons aged 65 and older. Osteoarthritis Cartilage.

[7] Felson DT, Naimark A, Anderson J, Kazis L, Castelli W, Meenan RF (1987). The prevalence of knee osteoarthritis in the elderly. The framingham osteoarthritis study. Arthritis Rheum.

[8] Jordan JM, Helmick CG, Renner JB, Luta G, Dragomir AD, Woodard J (2007). Prevalence of knee symptoms and radiographic and symptomatic knee osteoarthritis in African americans and Caucasians: the Johnston county osteoarthritis project. J Rheumatol.

[9] Miyasaka K, Daniel D, Stone M, Hirshman P (1991). The incidence of knee ligament injuries in the general population. Am J Knee Surg.

[10] Nielsen AB, Yde J (1991). Epidemiology of acute knee injuries: a prospective hospital investigation. J Trauma.

[11] Cella D, Riley W, Stone A, Rothrock N, Reeve B, Yount S (2010). The patient-reported outcomes measurement information system (promis) developed and tested its first wave of adult self-reported health outcome item banks: 2005–2008. J Clin Epidemiol.

[12] Bruce B, Fries JF, Ambrosini D, Lingala B, Gandek B, Rose M (2009). Better assessment of physical function: item improvement is neglected but essential. Arthritis Res Ther.

[13] Christodoulou C, Junghaenel DU, DeWalt DA, Rothrock N, Stone AA (2008). Cognitive interviewing in the evaluation of fatigue items: results from the patient-reported outcomes measurement information system (promis). Qual Life Res.

[14] Irwin DE, Varni JW, Yeatts K, DeWalt DA (2009). Cognitive interviewing methodology in the development of a pediatric item bank: a patient reported outcomes measurement information system (promis) study. Health Qual Life Outcomes.

[15] Amtmann D, Cook KF, Johnson KL, Cella D (2011). The promis initiative: involvement of rehabilitation stakeholders in development and examples of applications in rehabilitation research. Arch Phys Med Rehabil.

[16] Bruce B, Fries J, Lingala B, Hussain YN, Krishnan E (2013). Development and assessment of floor and ceiling items for the promis physical function item bank. Arthritis Res Ther.

[17] Daly J, Willis K, Small R, Green J, Welch N, Kealy M (2007). A hierarchy of evidence for assessing qualitative health research. J Clin Epidemiol.

[18] Coyne IT (1997). Sampling in qualitative research. Purposeful and theoretical sampling; merging or clear boundaries?. J Adv Nurs.

[19] Kuzel AJ, Crabtree BF, Miller WL (Ed). Doing qualitative research. Research methods for primary care, Vol. 3, (pp. 31-44). Thousand Oaks, CA, US: Sage Publications, Inc; 1992. pp. 276.

[20] Beatty PC, Willis GB (2007). Research synthesis: the practice of cognitive interviewing. Public Opinion Quarterly.

[21] Promis mission, vision & goals http://www.nihpromis.org/about/missionvisiongoals Accessed 3 Jan 2015

[22] Hefti F, Muller W, Jakob RP, Staubli HU (1993). Evaluation of knee ligament injuries with the ikdc form. Knee Surg Sports Traumatol Arthrosc.

[23] Hambleton RK, Swaminathan H, Rogers HJ (1991). Fundamentals of item response theory.

[24] Hung M, Clegg DO, Greene T, Saltzman CL (2011). Evaluation of the promis physical function item bank in orthopaedic patients. J Orthop Res.

