# Supplementary material for: Individuals with knee impairments identify items in need of clarification in the Patient Reported Outcomes Measurement Information System (PROMIS®) pain interference and physical function item banks – a qualitative study
Source: Health Qual Life Outcomes. 2016 May 11;14:77. doi: 10.1186/s12955-016-0478-7 (PMC4864902; doi:10.1186/s12955-016-0478-7)
Supplement: Additional file 2: — Item Results. (DOCX 26 kb) [file 12955_2016_478_MOESM2_ESM.docx]

| Table 1. PROMIS Pain Interference and Physical Function Items with Interview and Screening Results | | |  | |
| --- | --- | --- | --- | --- |
| Section 1. Items with content highly likely to be influenced by knee joint pathology | | | | |
| Item # | Item Stem | **Ultimate Item Fate** | **Item Bin** | |
| PAININ19 | How much did pain make it difficult to fall asleep? | Remains in Item Pool;  Removed from Cognitive Interview after 5 Interviews | 1 | |
| PAININ50 | How often did pain prevent you from sitting for more than 30 minutes? | Remains in Item Pool;  Removed from Cognitive Interview after 5 Interviews | 1 | |
| PAININ51 | How often did pain prevent you from sitting for more than 10 minutes? | Remains in Item Pool;  Removed from Cognitive Interview after 5 Interviews | 1 | |
| PAININ54 | How often did pain keep you from getting into a standing position? | Remains in Item Pool;  Removed from Cognitive Interview after 5 Interviews | 1 | |
| PAININ55 | How often did pain prevent you from sitting for more than one hour? | Remains in Item Pool;  Removed from Cognitive Interview after 5 Interviews | 1 | |
| PFA12 | Are you able to push open a heavy door? | Remains in Item Pool;  Removed from Cognitive Interview after 5 Interviews | 1 | |
| PFA16 | Are you able to dress yourself, including tying shoelaces and doing buttons? | Remains in Item Pool;  Removed from Cognitive Interview after 5 Interviews | 1 | |
| PFA30 | Are you able to step up and down curbs? | Remains in Item Pool;  Removed from Cognitive Interview after 5 Interviews | 1 | |
| PFA45 | Are you able to get out of bed into a chair? | Remains in Item Pool;  Removed from Cognitive Interview after 5 Interviews | 1 | |
| PFA51 | Are you able to sit on the edge of a bed? | Remains in Item Pool;  Removed from Cognitive Interview after 5 Interviews | 1 | |
| PFA52 | Are you able to tie your shoelaces? | Remains in Item Pool;  Removed from Cognitive Interview after 5 Interviews | 1 | |
| PFA56 | Are you able to get in and out of a car? | Remains in Item Pool;  Removed from Cognitive Interview after 5 Interviews | 1 | |
| PFA8 | Are you able to move a chair from one room to another? | Remains in Item Pool;  Removed from Cognitive Interview after 5 Interviews | 1 | |
| PFA9 | Are you able to bend down and pick up clothing from the floor? | Remains in Item Pool;  Removed from Cognitive Interview after 5 Interviews | 1 | |
| PFB11 | Are you able to wash dishes, pots, and utensils by hand while standing at a sink? | Remains in Item Pool;  Removed from Cognitive Interview after 5 Interviews | 1 | |
| PFB12 | Are you able to make a bed, including spreading and tucking in bed sheets? | Remains in Item Pool;  Removed from Cognitive Interview after 5 Interviews | 1 | |
| PFB17 | Are you able to put on and take off your socks? | Remains in Item Pool;  Removed from Cognitive Interview after 5 Interviews | 1 | |
| PFB25 | Are you able to push open a door after turning the knob? | Remains in Item Pool;  Removed from Cognitive Interview after 5 Interviews | 1 | |
| PFB40 | Are you able to stand up on tiptoes? | Remains in Item Pool;  Removed from Cognitive Interview after 5 Interviews | 1 | |
| PFB43 | Does your health now limit you in taking care of your personal needs (dress, comb hair, toilet, eat, bathe)? | Remains in Item Pool;  Removed from Cognitive Interview after 5 Interviews | 1 | |
| PFC33 | Are you able to run ten miles? | Remains in Item Pool;  Removed from Cognitive Interview after 5 Interviews | 1 | |
| PFC40 | Are you able to kneel on the floor? | Remains in Item Pool;  Removed from Cognitive Interview after 5 Interviews | 1 | |
| PFC45 | Are you able to get on and off the toilet? | Remains in Item Pool;  Removed from Cognitive Interview after 5 Interviews | 1 | |
| PFC46 | Are you able to transfer from a bed to a chair and back? | Remains in Item Pool;  Removed from Cognitive Interview after 5 Interviews | 1 | |
| PFC47 | Are you able to be out of bed most of the day? | Remains in Item Pool;  Removed from Cognitive Interview after 5 Interviews | 1 | |
| PFC7 | Are you able to run five miles? | Remains in Item Pool;  Removed from Cognitive Interview after 5 Interviews | 1 | |
| PFB7 | Does your health now limit you in doing strenuous activities such as backpacking, skiing, playing tennis, bicycling or jogging? | **Item Components incorporated into PFB51 Revisions** | 1 | |
| PFA42 | Are you able to carry a laundry basket up a flight of stairs? | Remains in Item Pool;  Removed from Cognitive Interview after 5 Interviews | 1 | |
| PFC6 | Are you able to walk a block on flat ground? | Remains in Item Pool;  Removed from Cognitive Interview after 5 Interviews | 1 | |
| PAININ12 | How much did pain interfere with the things you usually do for fun? | Remains in Item Pool;  Subjected to more than 5 Cognitive Interviews | | 1 |
| PAININ13 | How much did pain interfere with your family life? | Remains in Item Pool;  Subjected to more than 5 Cognitive Interviews | | 1 |
| PAININ17 | How much did pain interfere with your relationships with other people? | Remains in Item Pool;  Subjected to more than 5 Cognitive Interviews | | 1 |
| PAININ22 | How much did pain interfere with work around the home? | Remains in Item Pool;  Subjected to more than 5 Cognitive Interviews | | 1 |
| PAININ31 | How much did pain interfere with your ability to participate in social activities? | Remains in Item Pool;  Subjected to more than 5 Cognitive Interviews | | 1 |
| PAININ32 | How often did pain make you feel discouraged? | Remains in Item Pool;  Subjected to more than 5 Cognitive Interviews | | 1 |
| PAININ36 | How much did pain interfere with your enjoyment of social activities? | Remains in Item Pool;  Subjected to more than 5 Cognitive Interviews | | 1 |
| PAININ37 | How often did pain make you feel anxious? | Remains in Item Pool;  Subjected to more than 5 Cognitive Interviews | | 1 |
| PAININ38 | How often did you avoid social activities because it might make you hurt more? | Remains in Item Pool;  Subjected to more than 5 Cognitive Interviews | | 1 |
| PAININ40 | How often did pain prevent you from walking more than 1 mile? | Remains in Item Pool;  Subjected to more than 5 Cognitive Interviews | | 1 |
| PAININ46 | How often did pain make it difficult for you to plan social activities? | Remains in Item Pool;  Subjected to more than 5 Cognitive Interviews | | 1 |
| PAININ52 | How often was it hard to plan social activities because you didn't know if you would be in pain? | Remains in Item Pool;  Subjected to more than 5 Cognitive Interviews | | 1 |
| PAININ53 | How often did pain restrict your social life to your home? | Remains in Item Pool;  Subjected to more than 5 Cognitive Interviews | | 1 |
| PAININ56 | How irritable did you feel because of pain? | Remains in Item Pool;  Subjected to more than 5 Cognitive Interviews | | 1 |
| PAININ6 | How much did pain interfere with your close personal relationships? | Remains in Item Pool;  Subjected to more than 5 Cognitive Interviews | | 1 |
| PFB49 | Does your health now limit you in going for a short walk (less than 15 minutes)? | Remains in Item Pool;  Subjected to more than 5 Cognitive Interviews | | 1 |
| PFC12 | Does your health now limit you in doing two hours of physical labor? | Remains in Item Pool;  Subjected to more than 5 Cognitive Interviews | | 1 |
| PFC35 | Does your health now limit you in doing eight hours of physical labor? | Remains in Item Pool;  Subjected to more than 5 Cognitive Interviews | | 1 |
| PAININ1 | How difficult was it for you to take in new information because of pain? | Remains in Item Pool;  Subjected to more than 5 Cognitive Interviews | | 1 |
| PAININ10 | How much did pain interfere with your enjoyment of recreational activities? | Remains in Item Pool;  Subjected to more than 5 Cognitive Interviews | | 1 |
| PAININ11 | How often did you feel emotionally tense because of your pain? | Remains in Item Pool;  Subjected to more than 5 Cognitive Interviews | | 1 |
| PAININ14 | How much did pain interfere with doing your tasks away from home (e.g., getting groceries, running errands)? | Remains in Item Pool;  Subjected to more than 5 Cognitive Interviews | | 1 |
| PAININ16 | How often did pain make you feel depressed? | Remains in Item Pool;  Subjected to more than 5 Cognitive Interviews | | 1 |
| PAININ18 | How much did pain interfere with your ability to work (include work at home)? | Remains in Item Pool;  Subjected to more than 5 Cognitive Interviews | | 1 |
| PAININ20 | How much did pain feel like a burden to you? | Remains in Item Pool;  Subjected to more than 5 Cognitive Interviews | | 1 |
| PAININ24 | How often was pain distressing to you? | Remains in Item Pool;  Subjected to more than 5 Cognitive Interviews | | 1 |
| PAININ26 | How often did pain keep you from socializing with others? | Remains in Item Pool;  Subjected to more than 5 Cognitive Interviews | | 1 |
| PAININ29 | How often was your pain so severe you could think of nothing else? | Remains in Item Pool;  Subjected to more than 5 Cognitive Interviews | | 1 |
| PAININ3 | How much did pain interfere with your enjoyment of life? | Remains in Item Pool;  Subjected to more than 5 Cognitive Interviews | | 1 |
| PAININ34 | How much did pain interfere with your household chores? | Remains in Item Pool;  Subjected to more than 5 Cognitive Interviews | | 1 |
| PAININ35 | How much did pain interfere with your ability to make trips from home that kept you gone for more than 2 hours? | Remains in Item Pool;  Subjected to more than 5 Cognitive Interviews | | 1 |
| PAININ39 | How often did pain make simple tasks hard to complete? | Remains in Item Pool;  Subjected to more than 5 Cognitive Interviews | | 1 |
| PAININ42 | How often did pain prevent you from standing for more than one hour? | Remains in Item Pool;  Subjected to more than 5 Cognitive Interviews | | 1 |
| PAININ47 | How often did pain prevent you from standing for more than 30 minutes? | Remains in Item Pool;  Subjected to more than 5 Cognitive Interviews | | 1 |
| PAININ48 | How much did pain interfere with your ability to do household chores? | Remains in Item Pool;  Subjected to more than 5 Cognitive Interviews | | 1 |
| PAININ49 | How much did pain interfere with your ability to remember things? | Remains in Item Pool;  Subjected to more than 5 Cognitive Interviews | | 1 |
| PAININ5 | How much did pain interfere with your ability to participate in leisure activities? | Remains in Item Pool;  Subjected to more than 5 Cognitive Interviews | | 1 |
| PAININ8 | How much did pain interfere with your ability to concentrate? | Remains in Item Pool;  Subjected to more than 5 Cognitive Interviews | | 1 |
| PAININ9 | How much did pain interfere with your day to day activities? | Remains in Item Pool;  Subjected to more than 5 Cognitive Interviews | | 1 |
| PFA10 | Are you able to stand for one hour? | Remains in Item Pool;  Subjected to more than 5 Cognitive Interviews | | 1 |
| PFA11 | Are you able to do chores such as vacuuming or yard work? | Remains in Item Pool;  Subjected to more than 5 Cognitive Interviews | | 1 |
| PFA15 | Are you able to stand up from an armless straight chair? | Remains in Item Pool;  Subjected to more than 5 Cognitive Interviews | | 1 |
| PFA21 | Are you able to go up and down stairs at a normal pace? | Remains in Item Pool;  Subjected to more than 5 Cognitive Interviews | | 1 |
| PFA23 | Are you able to go for a walk of at least 15 minutes? | Remains in Item Pool;  Subjected to more than 5 Cognitive Interviews | | 1 |
| PFA31 | Are you able to get up off the floor from lying on your back without help? | Remains in Item Pool;  Subjected to more than 5 Cognitive Interviews | | 1 |
| PFA32 | Are you able to stand with your knees straight? | Remains in Item Pool;  Subjected to more than 5 Cognitive Interviews | | 1 |
| PFA37 | Are you able to stand for short periods of time? | Remains in Item Pool;  Subjected to more than 5 Cognitive Interviews | | 1 |
| PFA39 | Are you able to run at a fast pace for two miles? | Remains in Item Pool;  Subjected to more than 5 Cognitive Interviews | | 1 |
| PFA5 | Does your health now limit you in lifting or carrying groceries? | Remains in Item Pool;  Subjected to more than 5 Cognitive Interviews | | 1 |
| PFA53 | Are you able to run errands and shop? | Remains in Item Pool;  Subjected to more than 5 Cognitive Interviews | | 1 |
| PFA6 | Does your health now limit you in bathing or dressing yourself? | Remains in Item Pool;  Subjected to more than 5 Cognitive Interviews | | 1 |
| PFA7 | How much do physical health problems now limit your usual physical activities (such as walking or climbing stairs)? | Remains in Item Pool;  Subjected to more than 5 Cognitive Interviews | | 1 |
| PFB13 | Are you able to carry a shopping bag or briefcase? | Remains in Item Pool;  Subjected to more than 5 Cognitive Interviews | | 1 |
| PFB14 | Are you able to take a tub bath? | Remains in Item Pool;  Subjected to more than 5 Cognitive Interviews | | 1 |
| PFB24 | Are you able to run a short distance, such as to catch a bus? | Remains in Item Pool;  Subjected to more than 5 Cognitive Interviews | | 1 |
| PFB3 | Does your health now limit you in putting a trash bag outside? | Remains in Item Pool;  Subjected to more than 5 Cognitive Interviews | | 1 |
| PFB32 | Are you able to stand unsupported for 10 minutes? | Remains in Item Pool;  Subjected to more than 5 Cognitive Interviews | | 1 |
| PFB42 | Are you able to stand unsupported for 30 minutes? | Remains in Item Pool;  Subjected to more than 5 Cognitive Interviews | | 1 |
| PFB50 | How much difficulty do you have doing your daily physical activities, because of your health? | Remains in Item Pool;  Subjected to more than 5 Cognitive Interviews | | 1 |
| PFB54 | Does your health now limit you in going OUTSIDE the home, for example to shop or visit a doctor's office? | Remains in Item Pool;  Subjected to more than 5 Cognitive Interviews | | 1 |
| PFB8 | Are you able to carry two bags filled with groceries 100 yards? | Remains in Item Pool;  Subjected to more than 5 Cognitive Interviews | | 1 |
| PFC20 | Does your health now limit you in walking one hundred yards? | Remains in Item Pool;  Subjected to more than 5 Cognitive Interviews | | 1 |
| PFC31 | Are you able to reach into a low cupboard? | Remains in Item Pool;  Subjected to more than 5 Cognitive Interviews | | 1 |
| PFC34 | Does your health now limit you in walking several hundred yards? | Remains in Item Pool;  Subjected to more than 5 Cognitive Interviews | | 1 |
| PFC36 | Does your health now limit you in walking more than a mile? | Remains in Item Pool;  Subjected to more than 5 Cognitive Interviews | | 1 |
| PFC38 | Are you able to walk at a normal speed? | Remains in Item Pool;  Subjected to more than 5 Cognitive Interviews | | 1 |
| PFC39 | Are you able to stand without losing your balance for several minutes? | Remains in Item Pool;  Subjected to more than 5 Cognitive Interviews | | 1 |
| PFC41 | Are you able to sit down in and stand up from a low, soft couch? | Remains in Item Pool;  Subjected to more than 5 Cognitive Interviews | | 1 |
| PFC53 | Are you able to get in and out of bed? | Remains in Item Pool;  Subjected to more than 5 Cognitive Interviews | | 1 |
| PFC54 | Does your health now limit you in getting in and out of the bathtub? | Remains in Item Pool;  Subjected to more than 5 Cognitive Interviews | | 1 |
| **PFA1** | **Does your health now limit you in doing vigorous activities, such as running, lifting heavy objects, participating in strenuous sports?** | **Item Revised** | 1 | |
| PFA1.a | Does your health now limit you in doing vigorous activities including running, lifting heavy objects and participating in strenuous sports? | (Additional Item) |  | |
| PFA1.b | Does your health now limit you in doing vigorous activities? | (Additional Item) |  | |
| PFA1.c | Does your health now limit you in participating in sports that you would like to do? | (Additional Item) |  | |
| PFA1.d | Does your health now limit you in lifting heavy objects? | (Additional Item) |  | |
| **PFA13** | **Are you able to exercise for an hour?** | **Item Revised** | **1** | |
| PFA13.a | Are you able to exercise with your injured (body part) for an hour? | (Additional Item) |  | |
| **PFA14** | **Are you able to carry a heavy object (over 10 pounds)?** | **Item Revised** | **1** | |
| PFA14.a | Are you able to carry a heavy object? | (Additional Item) |  | |
| **PFA19** | **Are you able to run or jog for two miles?** | **Item Revised** | **1** | |
| PFA19.a | Are you able to run for 2 miles? | (Additional Item) |  | |
| PFA19.b | Are you able to jog for 2 miles? | (Additional Item) |  | |
| **PFA25** | **Are you able to do yard work like raking leaves, weeding, or pushing a lawn mower?** | **Item Revised** | **1** | |
| PFA25.a | Are you able to do yard work including raking leaves, weeding, pushing a lawn mower, and other similar activities? | (Additional Item) |  | |
| PFA25.b | Are you able to do yard work? | (Additional Item) |  | |
| **PFA29** | **Are you able to pull heavy objects (10 pounds) towards yourself?** | **Item Revised** | **1** | |
| PFA29.a | Are you able to pull heavy objects towards yourself? | (Additional Item) |  | |
| **PFA3** | **Does your health now limit you in bending, kneeling, or stooping?** | **Item Revised** | **1** | |
| PFA33.a | Are you able to exercise hard with your injured (body part) for half an hour? | (Additional Item) |  | |
| **PFA4** | **Does your health now limit you in doing heavy work around the house like scrubbing floors, or lifting or moving heavy furniture?** | **Item Revised** | **1** | |
| PFA4.a | Does your health now limit you in doing heavy work inside the house? | (Additional Item) |  | |
| **PFA41** | **Are you able to squat and get up?** | **Item Revised** | **1** | |
| PFA41.a | Are you able to squat like a baseball catcher and get back up? | (Additional Item) |  | |
| PFA41.b | Are you able to perform full squats in the gym with resistance? | (Additional Item) |  | |
| PFA41.c | Are you able to squat all the way down and get back up? | (Additional Item) |  | |
| **PFA47** | **Are you able to pull on trousers?** | **Item Revised** | **1** | |
| PFA47.a | Are you able to put on a pair of pants? | (Additional Item) |  | |
| **PFB1** | **Does your health now limit you in doing moderate work around the house like vacuuming, sweeping floors or carrying in groceries?** | **Item Revised** | **1** | |
| PFB1.a | Does your health now limit you in doing work around the house including vacuuming, sweeping floors and carrying in groceries? | (Additional Item) |  | |
| PFB1.b | Does your health now limit you in sweeping floors? | (Additional Item) |  | |
| PFB1.c | Does your health now limit you in carrying in groceries? | (Additional Item) |  | |
| PFB1.d | Does your health now limit you in doing moderate work around the house? | (Additional Item) |  | |
| **PFB10** | **Are you able to climb up five steps?** | **Item Revised** | **1** | |
| PFB10.a | Does your health now limit you in going up five steps? | (Additional Item) |  | |
| PFB10.b | Does your health now limit you in going down five steps? | (Additional Item) |  | |
| **PFB44** | **Does your health now limit you in doing moderate activities, such as moving a table, pushing a vacuum cleaner, bowling, or playing golf?** | **Item Revised** | **1** | |
| PFB44.a | Does your health now limit you in doing moderate activities? | (Additional Item) |  | |
| PFB44.b | Does your health now limit you in moving a table? | (Additional Item) |  | |
| PFB44.c | Does your health now limit you in pushing a vacuum cleaner? | (Additional Item) |  | |
| PFB44.d | Does your health now limit you in bowling? | (Additional Item) |  | |
| **PFB5** | **Does your health now limit you in hiking a couple of miles on uneven surfaces, including hills?** | **Item Revised** | **1** | |
| PFB5.a | Does your health now limit you in walking a couple of miles on uneven surfaces, including hills? | (Additional Item) |  | |
| **PFB51** | **Does your health now limit you in participating in active sports such as swimming, tennis, or basketball?** | **Item Revised** | **1** | |
| PFB51.a | Does your health now limit you in activities like swimming, cycling, or golf? | (Additional Item) |  | |
| PFB51.b | Does your health now limit you in activities like baseball, softball, or racquet sports? | (Additional Item) |  | |
| PFB51.c | Does your health now limit you in playing football, basketball, soccer, or other similar sports? | (Additional Item) |  | |
| **PFB9** | **Are you able to jump up and down?** | **Item Revised** | **1** | |
| PFB9.a | Are you able to jump up and down sufficiently to play sports? | (Additional Item) |  | |
| **PFC10** | **Does your health now limit you in climbing several flights of stairs?** | **Item Revised** | **1** | |
| PFC10.a | Does your health now limit you in going down several flights of stairs? | (Additional Item) |  | |
| PFC10.b | Does your health now limit you in going up several flights of stairs? | (Additional Item) |  | |
| **PFC13** | **Are you able to run 100 yards?** | **Item Revised** | **1** | |
| PFC13.a | Are you able to sprint 100 yards? | (Additional Item) |  | |
| PFC13.b | Are you able to jog 100 yards? | (Additional Item) |  | |
| PFC13.c | Are you able to run 100 yards? | (Additional Item) |  | |
| **PFC29** | **Are you able to walk up and down two steps?** | **Item Revised** | **1** | |
| PFC29.a | Are you able to walk up and down a set of 2 steps? | (Additional Item) |  | |
| **PFC32** | **Are you able to climb up 5 flights of stairs?** | **Item Revised** | **1** | |
| PFC32.a | Does your health now limit you in going down 5 flights of stairs? | (Additional Item) |  | |
| PFC32.b | Does your health now limit you in going up 5 flights of stairs | (Additional Item) |  | |
| **PFC37** | **Does your health now limit you in going up one flight of stairs?** | **Item Revised** | **1** | |
| PFC37.a | Does your health now limit you in going down one flight of stairs? | (Additional Item) |  | |
| **PFC56** | **Does your health now limit you in walking about the house?** | **Item Revised** |  | |
| PFC56.a | Does your health limit you in walking on the main floor of your house, not including stairs? | (Additional Item) |  | |
| Section 2. Items with content that may not be influenced by the knee joint. | | | | |
| PFA55 | Are you able to wash and dry your body? | Knee joint may be relevant to item (30%) | 2 | |
| PFC52 | Are you able to turn from side to side in bed? | Knee joint may be relevant to item (30%) | 2 | |
| PFB48 | Does your health now limit you in taking a shower? | Knee joint may be relevant to item (35%) | 2 | |
| PFB28 | Are you able to lift 10 pounds above your shoulder? | Knee joint may be relevant to item (35%) | 2 | |
| PFB34 | Are you able to change a light bulb overhead? | Knee joint may be relevant to item (35%) | 2 | |
| PFB39 | Are you able to reach and get down a 5 pound object from above your head? | Knee joint may be relevant to item (40%) | 2 | |
| PFA17 | Are you able to reach into a high cupboard? | Knee joint may be relevant to item (45%) | 2 | |
| PFA34 | Are you able to wash your back? | Knee joint may be relevant to item (50%) | 2 | |
| PFA49 | Are you able to bend or twist your back? | Knee joint may be relevant to item (60%) | 2 | |
| PFB31 | Are you able to open car doors? | Knee joint may be relevant to item (65%) | 2 | |
| PFA38 | Are you able to dry your back with a towel? | Knee joint may be relevant to item (65%) | 2 | |
| PFB22 | Are you able to hold a plate full of food? | Knee joint may be relevant to item (70%) | 3 | |
| PFC51 | Are you able to wipe yourself after using the toilet? | Knee joint deemed irrelevant to item (75%) | 3 | |
| PFB36 | Are you able to put on a pullover sweater? | Knee joint deemed irrelevant to item (80%) | 3 | |
| PFB18 | Are you able to shave your face or apply makeup? | Knee joint deemed irrelevant to item (80%) | 3 | |
| PFB33 | Are you able to remove something from your back pocket? | Knee joint deemed irrelevant to item (80%) | 3 | |
| PFC49 | Are you able to water a house plant? | Knee joint deemed irrelevant to item (85%) | 3 | |
| PFB27 | Are you able to tie a knot or a bow? | Knee joint deemed irrelevant to item (85%) | 3 | |
| PFB56 | Are you able to lift one pound (a full pint container) to shoulder level without bending your elbow? | Knee joint deemed irrelevant to item (85%) | 3 | |
| PFA18 | Are you able to use a hammer to pound a nail? | Knee joint deemed irrelevant to item (90%) | 3 | |
| PFA36 | Are you able to put on and take off a coat or jacket? | Knee joint deemed irrelevant to item (90%) | 3 | |
| PFA44 | Are you able to put on a shirt or blouse? | Knee joint deemed irrelevant to item (90%) | 3 | |
| PFA50 | Are you able to brush your teeth? | Knee joint deemed irrelevant to item (90%) | 3 | |
| PFB15 | Are you able to change the bulb in a table lamp? | Knee joint deemed irrelevant to item (90%) | 3 | |
| PFB26 | Are you able to shampoo your hair? | Knee joint deemed irrelevant to item (90%) | 3 | |
| PFA20 | Are you able to cut your food using eating utensils? | Knee joint deemed irrelevant to item (95%) | 3 | |
| PFA35 | Are you able to open and close a zipper? | Knee joint deemed irrelevant to item (95%) | 3 | |
| PFB16 | Are you able to press with your index finger (for example ringing a doorbell)? | Knee joint deemed irrelevant to item (95%) | 3 | |
| PFB21 | Are you able to pick up coins from a table top? | Knee joint deemed irrelevant to item (95%) | 3 | |
| PFB30 | Are you able to open a new milk carton? | Knee joint deemed irrelevant to item (95%) | 3 | |
| PFC43 | Are you able to use your hands, such as for turning faucets, using kitchen gadgets, or sewing? | Knee joint deemed irrelevant to item (95%) | 3 | |
| PFA22 | Are you able to open previously opened jars? | Knee joint deemed irrelevant to item (100%) | 3 | |
| PFA28 | Are you able to open a can with a hand can opener? | Knee joint deemed irrelevant to item (100%) | 3 | |
| PFA40 | Are you able to turn a key in a lock? | Knee joint deemed irrelevant to item (100%) | 3 | |
| PFA43 | Are you able to write with a pen or pencil? | Knee joint deemed irrelevant to item (100%) | 3 | |
| PFA48 | Are you able to peel fruit? | Knee joint deemed irrelevant to item (100%) | 3 | |
| PFA54 | Are you able to button your shirt? | Knee joint deemed irrelevant to item (100%) | 3 | |
| PFB19 | Are you able to squeeze a new tube of toothpaste? | Knee joint deemed irrelevant to item (100%) | 3 | |
| PFB20 | Are you able to cut a piece of paper with scissors? | Knee joint deemed irrelevant to item (100%) | 3 | |
| PFB23 | Are you able to pour liquid from a bottle into a glass? | Knee joint deemed irrelevant to item (100%) | 3 | |
| PFB29 | Are you able to lift a full cup or glass to your mouth? | Knee joint deemed irrelevant to item (100%) | 3 | |
| PFB37 | Are you able to turn faucets on and off? | Knee joint deemed irrelevant to item (100%) | 3 | |
| PFB41 | Are you able to trim your fingernails? | Knee joint deemed irrelevant to item (100%) | 3 | |
|  |  |  |  | |
|  |  |  |  | |

- Item Bin 1: Content highly likely to be influenced by knee joint pathology.
- Item Bin 2: Content where knee joint pathology has questionable influence on the response.
- Item Bin 3: Content not likely to be influenced by knee joint pathology.
- Items removed after 5 interviews were deemed to be inherently clear.
- Items unchanged after more than 5 interviews did not present with significant clarity issues.
- Items which were revised presented significant clarity issues and were altered to improve understanding.
- Items from Bins 2 and 3 deemed “not relevant” by fewer than 75% of respondents were subjected to Cognitive Interviews.
- Items from Bin 3 deemed “not relevant’ by 75% of participants or greater were considered to not be influenced by the knee joint and should not be included in CATs or Short Forms for individuals with knee joint pathology.
